# Tethered Cord Syndrome and Its Association With Cardiovascular and Other Anomalies: Insights From a Prospective Observational Cohort of 60 Cases

**DOI:** 10.7759/cureus.92644

**Published:** 2025-09-18

**Authors:** Neeraj Prasad, Manisha Gupta, Rajendra Singh, Abhishek Kumar, Vipin Khunte, Akshay Khunte, Ramesh Gurjar

**Affiliations:** 1 Department of Neurosurgery, Super Specility Hospital, Chhattisgarh Institute of Medical Sciences, Koni, Bilaspur, IND; 2 Department of Neurology, Super Speciality Hospital, Chhattisgarh Institute of Medical Sciences, Koni, Bilaspur, IND; 3 Department of General Surgery, Chhattisgarh Institute of Medical Sciences, Bilaspur, IND; 4 Department of Cardiology, Super Speciality Hospital, Chhattisgarh Institute of Medical Sciences, Bilaspur, IND; 5 Department of Neurosurgery, Gajra Raja Medical College, Gwalior, IND; 6 Department of Internal Medicine, Pandit Jawahar Lal Nehru Memorial Medical College, Raipur, IND

**Keywords:** adhesion, cardiac anomalies, detethering, multidisciplinary care, tethered cord syndrome, urinary function

## Abstract

Background

A tethered cord is a neurological condition characterized by an abnormal attachment or tension of the spinal cord, which restricts its movement within the spinal canal. This dysfunction can lead to progressive neurological, orthopaedic, and urological symptoms. The clinical presentation and outcomes can be highly variable and associated with other system abnormalities, necessitating careful diagnosis and individualized management strategies. This study aims to define the clinical presentation along with associated cardiac and other spectrum, surgical outcomes, and the necessity for multidisciplinary management in tethered cord syndrome (TCS).

Materials and methods

A prospective cohort of children and young adults (age 1 year to 25 years) with clinically and radiologically confirmed TCS on MRI underwent baseline clinical, imaging, and echocardiographic evaluation. All received individualized detethering surgery, followed by standard postoperative assessments, including neurological, urological (with urodynamics and quality-of-life evaluations), and cardiac assessments. Patients had longitudinal follow-up for functional outcomes, complications, and recurrence, with strong multidisciplinary involvement throughout.

Results

The cohort demonstrated a male predominance with a broad spectrum of systemic anomalies. Cardiac anomalies were identified in 21 (35%) of patients via routine echocardiographic screening. Outcomes were analysed using descriptive statistics, and pre-/post-operative comparisons employed paired t-tests or Wilcoxon signed-rank tests as appropriate for continuous variables. Categorical outcomes were assessed using the chi-squared test. Reported improvement rates (urinary, motor) reached statistical significance (p < 0.05). The median duration of follow-up was 16 months (range 12-24 months). Quality of life (QoL) was measured using the PedsQL™ pediatric quality of life inventory, a validated tool for children, and the International Prostate Symptom Score for young adults. Multidisciplinary management involved neurologists, neurosurgeons, urologists, orthopedic surgeons, and cardiologists, with scheduled follow-ups at three months, six months, and one year in the first year and semi-annual visits thereafter. Early surgical intervention led to significant neurological and functional improvements (p = 0.02), particularly in urinary function (an 81.25% improvement) and motor deficits (a 90% improvement). Quality of life measures corroborated these findings. Each specialty contributed to integrated assessment and individualized care planning.

Conclusion

Tethered cord is a complex, predominantly paediatric syndrome with multisystem involvement requiring more than neurosurgical attention. Routine cardiac and renal screening should be integrated into diagnostic protocols to uncover occult comorbidities. Early surgical management yields meaningful improvements in function and quality of life. Long-term, multidisciplinary care models are critical to address the holistic needs of TCS patients. Major limitations of the study include the single-centre design, moderate cohort size, limited generalizability to diverse populations, and lack of genetic testing. Future multicenter studies with larger cohorts are needed to refine genotype-phenotype correlations and optimize management strategies.

## Introduction

Tethered cord syndrome (TCS) is a congenital disorder marked by abnormal spinal cord fixation, resulting in neurological and musculoskeletal impairments. While primarily affecting the spine, TCS is increasingly linked to a wider array of congenital anomalies, including those of the cardiovascular system. Notably, studies show a connection between TCS and congenital heart disease (CHD), especially cardiac septal defects. A major U.S. epidemiological study using the Nationwide Inpatient Sample (NIS) database analyzed 13,470 TCS hospital discharges (2003-2012) and found that about 12.6% of patients had at least one congenital anomaly [1]. The spinal, urinary, gastrointestinal, and cardiac systems were predominantly involved. Among additional anomalies of TCS cases, spinal anomalies were most frequent (24.48%); cardiac anomalies, mainly atrial and ventricular septal defects, occurred in 6.27% of cases [1]. Urinary malformations were seen in 5.37%. Multisystem anomalies, such as overlapping gastrointestinal and cardiac defects, were common, present in 4.55% of the cohort, underscoring TCS’s multisystem impact. The TCS-CHD co-occurrence likely originates from shared embryological insults during early mesodermal and neuroectoderm development. Mechanistically, disruptions in the neurulation phase and somitogenesis due to defects in signalling pathways (FGF, BMP, SHH) implicated in axial and cardiovascular patterning can lead to temporospatial overlap of these developmental processes, resulting in concurrent spinal and cardiac malformations. This multisystem involvement is also observed in syndromes such as VACTERL association. VACTERL is a term utilized to describe the aggregation of findings of vertebral malformations, anal atresia, cardiac anomalies, tracheoesophageal fistulas, renal anomalies, and limb malformations. Such syndromic features are reported in about 13.45% of TCS patients [2]. The incidence of TCS in patients with VACTERL is clinically significant, and physicians must be cautious in performing a comprehensive assessment of these patients for TCS to intervene and treat this condition. Although few large Indian studies focus directly on TCS-CHD links, Indian data reveal nearly 47% of congenital scoliosis patients have intraspinal issues, with tethered cord most common [3]. However, Indian data specifically quantifying CHD in TCS patients are lacking, revealing a significant knowledge gap. Recent Indian data reveal a lower reported incidence of TCS compared to global studies, likely due to underdiagnosis and referral patterns. Regional studies from tertiary centers estimate incidence at 1.8 to 2.7 per 10,000 births [3]. However, comprehensive national registry data on TCS-CHD associations are lacking, which restricts generalizability and impedes the development of locally relevant screening and management guidelines. Cardiovascular anomalies in TCS patients significantly impact perioperative risk, long-term outcomes, and management. Even asymptomatic defects like septal defects or mild valvular abnormalities can cause cardiac stress and complications during anaesthesia or surgery, including arrhythmias and heart failure [4]. Limited large-scale population data on multisystem anomalies in TCS hamper the development of evidence-based, multidisciplinary screening and management protocols. This study aims to address this gap by providing a detailed analysis to inform clinical care pathways better.

## Materials and methods

Study design

This was a prospective observational study conducted at a tertiary care center located in the central part of India, serving a predominantly tribal population. The study duration was from December 2018 to December 2023, extended due to the COVID-19 pandemic. The sample size was calculated to be approximately 64, based on a 12% population proportion of TCS patients with multisystem anomalies, as reported in a major U.S. epidemiological study by Horn et al. [1], using a 95% confidence level and an 8% margin of error. Despite pandemic delays, 60 patients were ultimately included.

Objectives

The objective of this study was to investigate the clinical, pathological, and radiological characteristics of TCS in children and young adults, to evaluate the surgical outcomes in affected patients, and to specifically examine the cardiac anomalies, along with other associated anomalies, in this population.

Inclusion criteria

The inclusion criteria were standardized and explicitly defined for this prospective cohort study. Children and young adults aged 1 year to 25 years with clinically and radiologically confirmed TCS on MRI were enrolled. Clinically, participants exhibited neurological deficits linked to lower spinal cord dysfunction such as motor or sensory symptoms, bowel or bladder issues, or progressive neurological decline. Diagnosis was confirmed radiologically by MRI, revealing a low-lying conus medullaris, a thickened filum terminale, or other tethering lesions, such as lipomas or diastematomyelia [5]. Only patients meeting both clinical and MRI criteria were enrolled, in line with established TCS diagnostic standards for research and clinical practice.

Exclusion criteria

Exclusion criteria were based on surgical considerations following an initial review of all TCS cases. Patients with severe co-morbidities that posed high surgical or anaesthetic risk (such as uncontrolled cardiac or respiratory disease, or active infections) were excluded. Other exclusion criteria were patients with normal imaging and no symptoms, asymptomatic incidental tethered cords, and those with severe, irreversible neurological deficits unlikely to benefit from surgery. Cases with a previous operated history of meningomyelocele, post-traumatic or infective spinal scarring, previous spinal surgeries resulting in scar tissue, or non-congenital causes of tethering (including tumours or inflammatory conditions) were also excluded to focus on congenital TCS. Non-cooperative patients unable to comply with pre- and postoperative care or follow-up were excluded to ensure safety and reliable outcomes. These criteria were designed to optimize surgical candidacy and enhance the validity of the study.

Ethical and regulatory approvals

This study was approved by both the Institutional Thesis Review Committee and the Institutional Ethics Committee. All patients received detailed information regarding the study's nature, objectives, benefits, and risks, after which written informed consent was obtained. The study adhered to the Declaration of Helsinki (2013 revision), ensuring that patient rights, safety, and well-being were maintained throughout [6]. Participation was entirely voluntary and involved no financial burden for patients.

Preoperative clinical and radiological assessment

**Figure 1:**
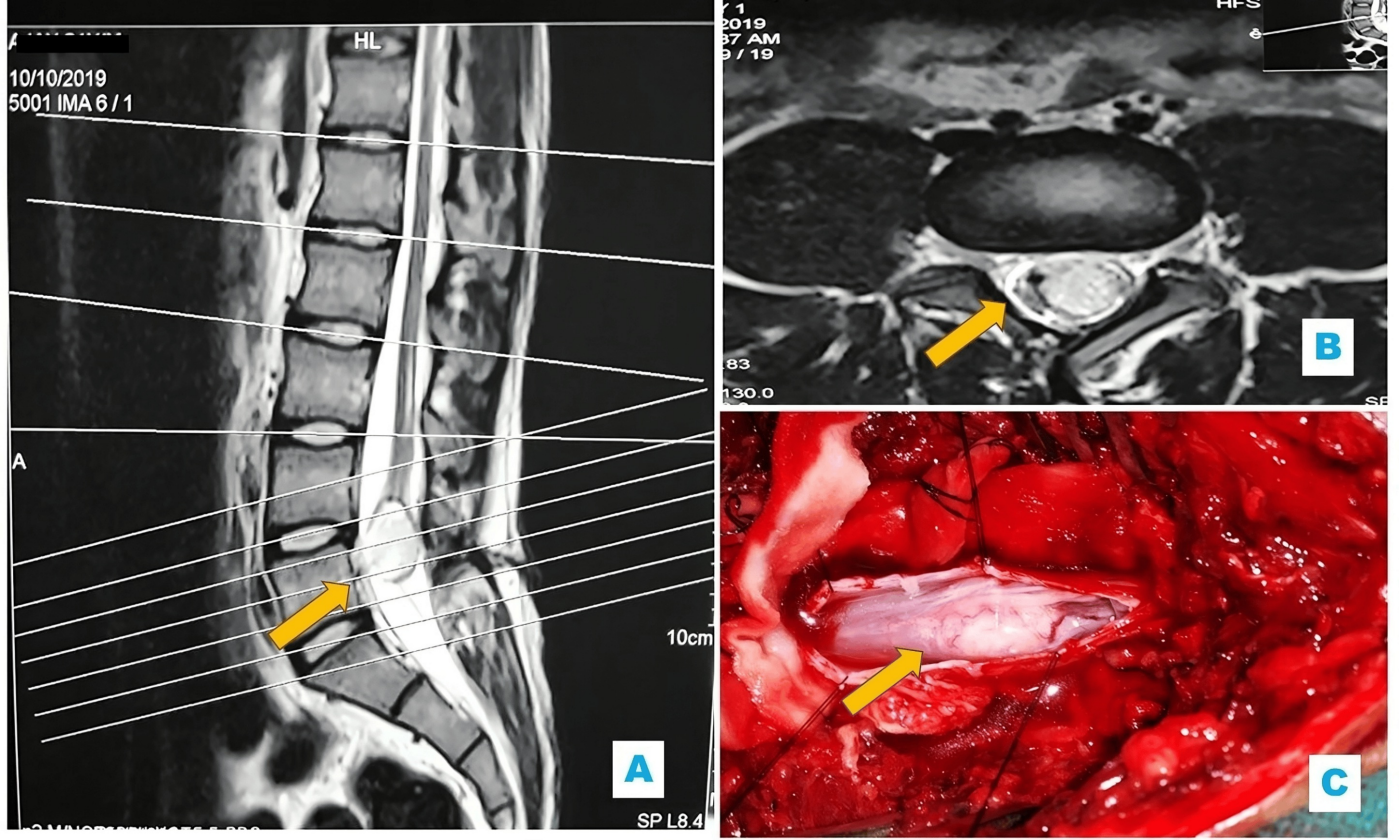
(A) Sagittal T2-weighted MRI demonstrating a well-defined dermoid cyst (indicated by arrow) at the level of the lower lumbar spine, associated with a low-lying conus medullaris. (B) Axial T2-weighted MRI at the same level, showing the dermoid cyst adjacent to the conus medullaris (arrow). (C) Intraoperative photograph revealing the exposed conus dermoid (arrow) following surgical exposure

All patients underwent a thorough preoperative assessment, starting with detailed histories covering prenatal to postnatal periods. Comprehensive physical and neurological exams identified focal deficits. The diagnostic workup included imaging-MRI, CT, X-ray, and ultrasonography (USG)-based on patient affordability, with MRI serving as the gold standard for TCS [5]. MRI specifically assessed conus medullaris level and filum terminale characteristics; patients with a thick or normal filum and a conus below L1-L2 (filum >2mm at L5-S1) were eligible for inclusion (Figure 1).

Two specialists (one radiologist and one neurosurgeon) performed all MRI image analyses independently to minimize inter-observer variability. CT was reserved for suspected split cord malformations. Abdominal USG helped identify associated congenital anomalies and measure post-void residual (PVR) urine. For infants, spinal USG was a valuable first-line screening tool for occult dysraphism due to its accessibility and imaging quality, although its sensitivity declines with age. PVR was measured by bladder USG, scanner, or catheterization (gold standard), ideally within five minutes post-voiding to avoid overestimation [5]. In adults, a persistent PVR above 50 ml was clinically important, though thresholds varied with age and guideline recommendations [7]. Chang et al. defined elevated PVR as >30 ml or >21% bladder capacity (BC) in children <6 years; >20 ml or 15% BC in ages 7-12 years; and >30 ml (11% BC) in 12-18 years [8]. This definition has been adopted by the International Children's Continence Society (ICCS) since 2015 [8]. Every patient also underwent echocardiography by a cardiologist trained in paediatric echocardiography preoperatively to detect any associated cardiac anomalies, supporting multidisciplinary planning. Echocardiographic evaluation followed a structured protocol using transthoracic echocardiography and transoesophageal echocardiography if required. Cardiac anomalies were classified according to standard nosology: major shunt & valvular lesions (e.g., moderate to large ASD, VSD, or PDA and moderate to severe MR, AR, MS, AS) or minor (e.g., patent foramen ovale, mild chamber enlargement, small shunt or mild valvular lesions) [9]. Findings were considered clinically significant if they required intervention, altered perioperative management, or had prognostic implications; otherwise, they were deemed incidental.

Surgical technique

The surgical procedure for TCS began with standard preparation and draping, including preoperative antibiotics. A midline skin incision was made, and paraspinal muscles were retracted to expose the spine. Depending on the pathology, laminotomy with laminoplasty or laminectomy (one level above and below the lesion) was performed. The dura was opened one level above the lesion to identify the normal cord, then the dissection proceeded caudally. Under microscopic guidance, tethering elements were carefully separated and excised. Neural tissue was distinguished from fibrous or fatty lesions like lipomas, which were debulked in pieces to avoid injury. In split cord malformations, all tethering tissues, including mesenchymal or bony spurs, were removed to free the hemicords. Adhesions such as arachnoid bands were delicately dissected. Dermoid cysts or sinus tracts were fully excised to prevent recurrence (Figure 1). The exposed tethered tissues were sutured with fine Vicryl to reduce adhesion formation. The filum terminale was inspected and sectioned at the lowest level possible after freeing adhesions, guided by texture, orientation, and low-current electrical stimulation. Intraoperative neuro monitoring was not done as the facility for the same was not available. This meticulous microsurgical approach aimed to achieve complete cord untethering while preserving neurological function.

Post-operative care and follow-up

Postoperative management included flat-bed rest for 24 hours to reduce CSF leak and headache risk, 24 hours of prophylactic antibiotics (no routine extension), and regular vital sign monitoring. Foley catheters were removed after stabilization, and bladder function was assessed via post-void residual measurement, given frequent postoperative retention. Common complications included wound infections, CSF leaks, and seroma formation, managed per clinical protocols. Single blinding was applied for clinical outcomes assessment, and they were categorized as improved, unchanged, or worsened based on symptomatic assessment of the patients post-surgery and comparing their dynamic nature with pre-op, as validated by the study of Kang et al. [10]. Recurrence and adhesion formation were determined by clinical criteria (worsening neurological deficit, new-onset symptoms) and contrast-enhanced MRI imaging to document anatomical changes or retethering. Multidisciplinary management involved neurologists, neurosurgeons, urologists, orthopedic surgeons, and cardiologists, with scheduled follow-ups at three months, six months, and one year in the first year and semi-annual visits thereafter. QoL for younger children was measured with the PedsQL™ pediatric inventory, covering physical, emotional, social, and school functioning. Baseline and follow-up scores tracked domains and overall status [11]. Older children and young adults also completed an IPSS item (0-6 scale) for urinary symptom impact; lower scores indicated better status [12]. Uroflowmetry (Qmax, PVR) was performed at each visit.

Statistical analysis

Data collected during the study were entered into Microsoft Excel™ (Microsoft Corporation, Redmond, USA) and analyzed using IBM SPSS Statistics for Windows, Version 27 (Released 2019; IBM Corp., Armonk, New York, United States). Descriptive statistical methods were employed to summarize the findings. Continuous variables were expressed as mean ± standard deviation (SD), while categorical variables were presented as frequencies and percentages. A P-value less than 0.05 was considered statistically significant.

## Results

The study cohort consisted of 60 patients, with the majority (60%) aged between 0 and 5 years. The next largest age group was 6 to 10 years (20%), followed by 16 to 20 years (10%), 11 to 15 years (6.7%), and a small proportion (3.3%) was older than 20 years. Regarding gender distribution, 65% of the patients were male, while 35% were female. Various anomalies, including cardiovascular anomalies associated with TCS cases, are listed in Table 1.

**Table 1:** Various anomalies along with cardiovascular anomalies associated with tethered cord syndrome cases in the present study population ^#^Few patients had overlapping lesions and features*.* TCS, tethered cord syndrome; CHD, congenital heart disease

Overall Anomaly	No. of Patients (n=60)#	Percentage (%)	Remarks
Any Associated Anomaly	24	40.0	At least one additional anomaly in TCS patients
Spinal Anomalies	19	31.7	Most frequent anomaly: Spina bifida is the most common specific form
Spina Bifida (specific spinal anomaly)	8	13.3	Frequently reported specific vertebral anomaly
VACTERL Association (Multisystem Anomaly)	8	13.3	Cluster of vertebral, cardiac, renal, and other anomalies
Isolated Cardiac Anomalies	7	11.7	Includes atrial and ventricular septal defects, the most common cardiac association
Gastrointestinal + Cardiac Combination	6	10.0	A combination of GI and cardiac anomalies in some patients
Urinary + Cardiac Combination	5	8.3	A combination of urinary and cardiac anomalies was observed
Cardiac Anomaly	No. of Patients (n=21)#	Percentage (%)	Remarks
Ventricular Septal Defect	8	38.0	Most common overall, isolated, or part of complex CHDs
Atrial Septal Defect	7	33.3	Frequently diagnosed in later infancy or childhood
Patent Ductus Arteriosus	5	23.8	More common in infants
Pulmonary Stenosis	4	19.0	May be isolated or part of syndromes
Tetralogy of Fallot	3	14.3	The most common cyanotic congenital heart defect
Atrioventricular Septal Defect	2	9.5	More frequent in children with chromosomal abnormalities
Coarctation of the Aorta	1	4.8	Often associated with genetic syndromes
Bicuspid Aortic Valve	1	4.8	May be asymptomatic in childhood, with clinical impact later

**Figure 2:**
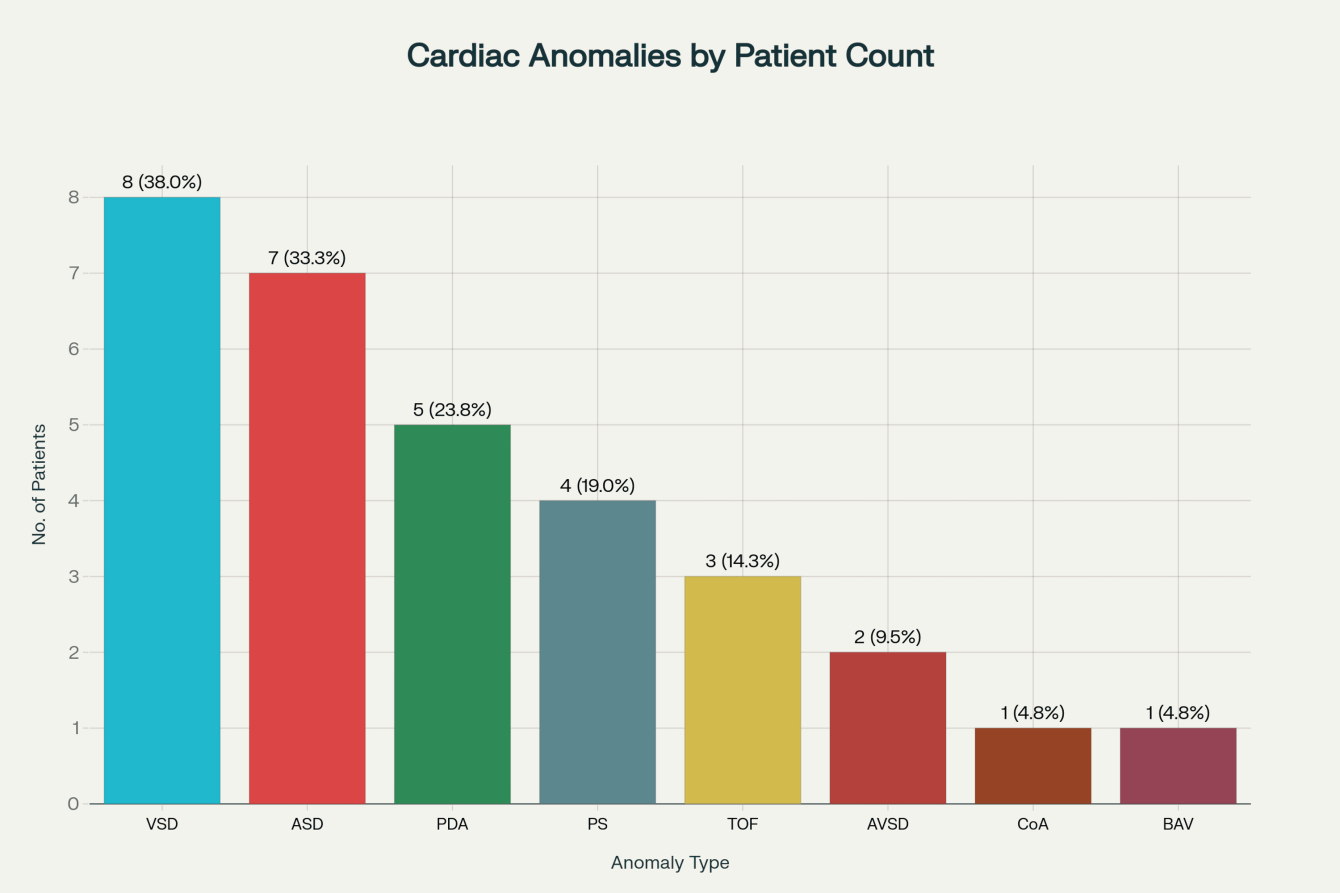
Bar chart showing the distribution of cardiac anomalies in TCS patients (n=21). Few patients had overlapping lesions and features VSD, ventricular septal defect; ASD, atrial septal defect; PDA, patent ductus arteriosus; PS, pulmonary stenosis; TOF, tetralogy of Fallot; CoA, coarctation of the aorta; AVSD, atrioventricular septal defect; BAV, bicuspid aortic valve.

Associated anomalies occurred in 40.7% of patients, primarily in the spinal region (24.48%). Among 21 cardiac cases, seven patients had an isolated cardiac defect, while 14 patients had combined cardiac and other system defects. In overall cardiac anomalies, the most frequent was a ventricular septal defect (38%), followed by an atrial septal defect (33.3%). Few patients had more than one cardiac anomaly (Figure 2).

The mean age of the cohort with no associated anomalies was 12.6 ± 2.3 years, with a male-to-female ratio of 1.3:1. In the cohort with associated anomaly, the mean age was 9.4 ± 1.7 years, and to male-to-female ratio was 1.25:1. Overview of clinical presentation and symptom prevalence among the study population is shown in Table 2.

**Table 2:** Overview of clinical presentation and symptom prevalence among the study population

Clinical Symptom	No. of Patients	Percentage (%)
Urinary Incontinence	34	56.7
Swelling in the Lower Back	33	55.0
Fecal Incontinence	23	38.3
Gait Abnormalities	12	20.0
Bilateral Lower Limb Weakness	12	20.0
Lower Limb Pain	19	31.6
Heart Failure	5	8.3
Sensory Loss	7	11.6
Foot Deformities (Clubfoot, Shortening)	5	8.3
Impotence	3	5.0
Tuft of Hair	2	3.3
Skin Dimple	2	3.3
Other Unspecified Symptoms	1	1.6

The most frequent clinical symptoms included urinary incontinence (56.7%) and swelling in the upper or lower back (55%). The type of spinal dysraphism associated with tethered cord in the study population and the type of operation done in the present study are shown in Table 3. Sacral lipomyelomeningocele is the most common spinal dysraphism (35%), followed by dermal sinus (16.7%) and lumbar meningomyelocele (11.7%), with lipomyelomeningocele types predominating overall. Few patients had overlapping lesions and features.

**Table 3:** Types of spinal dysraphism associated with tethered cord in the study population and their operative description ^#^Few patients had overlapping lesions and features.

Type of Spinal Dysraphism#	No. of Total Patients (n=60) (%)	No. of Operated Patients (n=45) (%)	Operation Description
Sacral Lipomyelomeningocele	21 (35%)	18 (40%)	Excision of Lipoma with Division of Filum Terminale
Dermal Sinus	10 (16.7)	8 (17.7%)	Excision of the Sinus with Division of Adhesions
Lumbar Meningomyelocele	14 (23.3%)	12 (26.6%)	Excision of Meningomyelocele with Excision of Filum Terminale
Cervical Meningomyelocele	5 (8.3)	3 (6.6%)	Excision of Meningomyelocele with Division of Adhesion
Thickened Filum Terminale	4 (6.7)	3 (6.6%)	Excision of Filum Terminale
Lumbar Lipomyelomeningocele	4 (6.7)	2 (4.4%)	Excision with Repair
Diastematomyelia	2 (3.3)	1 (2.2)	Laminectomy with Excision of Bony Spur with Excision of Filum
Dorsal Meningomyelocele	2 (3.3)	1 (2.2%)	Excision with Division of Adhesion
Conus Dermoid	1 (1.6)	1 (2.2)	Laminectomy with Near-Total Excision and Excision of the Filum Terminale

**Figure 3:**
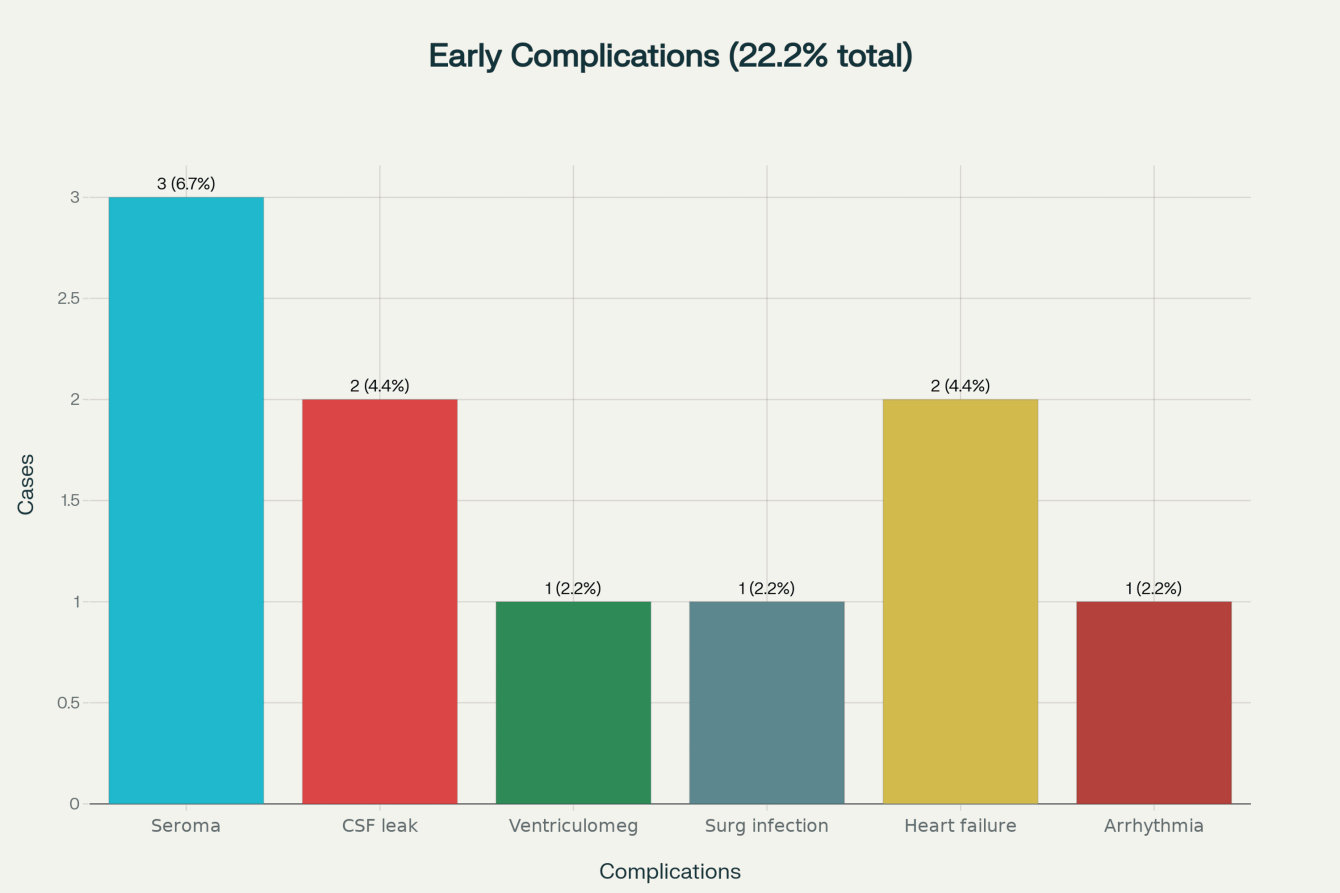
Bar chart showing early complications after surgery in the study population

Following detailed pre-operative clinical and radiological assessment and applying exclusion criteria, 45 patients were taken up for surgery. Major lesions from cardiac anomaly were excluded and referred to the paediatric cardiac center for further management. However, small ASD and VSD patients were included in the surgery after due cardiology fitness and consent. Among 45 patients, early complications included seroma formation and CSF leak (pseudo meningocele) in three and two cases, respectively, post-operative ventriculomegaly and surgical site infection in one case each, heart failure in two cases, and arrhythmia in one case, totalling 10 complications (22.2%) (Figure 3). Evan’s ratio was used after CT brain for diagnosis of ventriculomegaly and then shunting was done postoperatively. All patients' complications were resolved within one month.

There was no mortality. Heart failure was clinically defined using European Society of Cardiology (ESC) Guidelines for the diagnosis and treatment of acute and chronic heart failure [13]. It was supplemented by elevated levels of the biomarkers NT-proBNP and high-sensitivity troponin (hs-troponin), which significantly aid diagnosis and prognostication [14]. Age was a strong independent risk factor: In younger children, the odds ratio for heart failure increases by 1.3, with a 95% confidence interval of 1.2 to 1.4. There was no significant difference in heart failure risk among male and female patients (OD-1.08, CI-0.9-1.14). Heart failure was typically managed with diuretics, vasodilators, oxygen, and optimization of pre-existing cardiac medications, alongside monitoring fluid balance and renal function [15]. Arrhythmia was confirmed using a combination of clinical assessment and electrocardiographic/Holter monitoring (to detect arrhythmias like atrial fibrillation). It was managed by correcting reversible factors, using beta-blockers, antiarrhythmics for rate or rhythm control, and anticoagulation for atrial fibrillation (as duration exceeds 48 hours and bleeding risk allowed) [16]. Follow-up outcomes were analyzed based on the initial presenting symptoms and their change over time and are shown in Table 4.

**Table 4:** Clinical outcomes of the initial presenting symptoms after the follow-up duration

Sign/Symptom	Improved	Stable	Worse	Recurrence	Unknown	Total Patients
Urinary Dysfunction	18 (78.3%)	3 (13.0%)	0	0	2 (8.7%)	23
Motor Deficit	15 (88.2%)	2 (11.8%)	0	0	0	17
Bowel Dysfunction	9 (60.0%)	4 (26.7%)	0	0	2 (13.3%)	15
Sensory Deficit	6 (85.7%)	0	1 (14.3%)	0	0	7
Pain	9 (81.8%)	0	0	2 (18.2%)	0	11
Sexual Dysfunction	1 (50%)	0	0	0	1 (50%)	1

The median duration of follow-up was 16 months (range 12-24 months). Table 4 shows that most patients experienced improvement in symptoms, with urinary dysfunction improving in 78.3% and motor deficits in 88.2%. Stability was noted in a smaller portion, while worsening and recurrence were rare. Unknown outcomes were minimal except in sexual dysfunction, which had only one case with an unknown status. However, sexual dysfunction in this study cohort is likely to be underrepresented due to the younger age group. Urological parameters during follow also significantly improved (Table 5): Qmax 7.1→11.9 mL/s (p=0.0012), PVR 165.5→48.5 mL (p=0.0005), IPSS 23.5→13.8 (p=0.0031), QOL 4.5→1.9 (p=0.0014) over one year. There was one case of retethering. Age at surgery (< 5 years v/s ≥5 years) as a subgroup analysis found to be statistically significant for QOL (p=0.002).

**Table 5:** Functional urological outcomes analyzed based on initial presentation & over time during follow-up with ANOVA summary Qmax, maximum urinary flow rate; PVR, post-void residual urine volume; IPSS, International Prostate Symptom Score; QOL, quality of life score.

Outcome	Pre-operation Mean ± SD	95% CI	3 Months Mean ± SD	95% CI	6 Months Mean ± SD	95% CI	1 Year Mean ± SD	95% CI	F-value	p-value
Qmax (mL/s)	7.1 ± 1.9	6.4–7.8	12.6 ± 3.0	11.5–13.7	12.3 ± 2.9	11.2–13.4	11.9 ± 2.7	10.9–12.9	5.85	0.0012
PVR (mL)	165.5 ± 24.8	156.6–174.4	50.1 ± 14.6	44.9–55.3	52.7 ± 13.9	47.8–57.6	48.5 ± 12.7	44.1–52.9	7.23	0.0005
IPSS Score	23.5 ± 3.4	22.3–24.7	14.1 ± 2.7	13.1–15.1	14.3 ± 3.0	13.2–15.4	13.8 ± 2.8	12.7–14.9	4.97	0.0031
QOL Score	4.5 ± 0.6	4.3–4.7	2.1 ± 0.6	1.9–2.3	2.0 ± 0.5	1.8–2.2	1.9 ± 0.6	1.7–2.1	6.12	0.0014

## Discussion

Our prospective series of 60 patients provides a valuable window into the complex clinical spectrum, demographics, associated anomalies, management, and post-surgical outcomes in TCS, with a unique focus on cardiovascular associations as given by Venkataramana et al., an area with sparse systematic data [17]. Unlike Venkataramana et al., who focused on urogenital and anorectal anomalies in TCS and surgical management of tethered filum lipoma, our study systematically examines cardiovascular associations, providing new prevalence data, clinical correlations, and outcomes. This highlights the need for routine cardiovascular screening in TCS, supporting early detection and multidisciplinary care alongside neurological and urological management, in line with current guidelines for preserving function and preventing progression.

Patient demographics and baseline characteristics

Our cohort’s demographic analysis shows a clear pediatric predominance, with 60% aged 0-10 years, confirming TCS as predominantly a childhood congenital disorder, consistent with global findings [18]. Early onset is typically linked to visible cutaneous markers, neurological deficits, or urogenital symptoms that prompt caregiver and pediatric clinician recognition. However, 20% presented during adolescence and early adulthood. Delayed presentation of TCS in adolescents or adults often leads to worse prognosis and less optimal surgical outcomes because progressive neurological and urological deficits may become irreversible with prolonged cord tension and damage. Gender distribution shows marked male predominance (65%), aligning with higher congenital spinal dysraphism rates in males, though underlying causes remain unclear [19]. Proposed explanations include sex-linked genetic and hormonal factors during neural tube development or cohort methodology differences. Cultural factors such as lack of awareness, pessimistic counseling by physicians, preference for traditional medicine, and gender-related social considerations (e.g., treatment sought before marriage in females) contribute to late diagnosis in some populations. Meta-analysis by McVeigh reports a male-to-female ratio of 1.6:1 in congenital TCS, but in some cohorts, the ratio is more balanced [20]. Importantly, sex differences in outcomes go beyond prevalence: males may have earlier symptom onset and more comorbidities, yet females often face higher post-surgical infection risks such as UTIs, possibly due to anatomical differences, influencing recovery and postoperative care needs [20].

TCS and associated cardiac and other anomalies

Analysis of associated anomalies in our cohort highlights the syndromic nature of TCS, with 40.0% of patients having at least one additional congenital anomaly. This high prevalence confirms that TCS rarely occurs in isolation and should prompt clinicians to consider broader developmental field defects during evaluation. Spinal anomalies were present in 31.7%, with spina bifida the most common vertebral defect (13.3%), consistent with 21-30% spinal dysraphism co-prevalence among TCS patients reported by Horn et al. [1]. Occult spina bifida can remain clinically silent until TCS symptoms such as neurological deficits or bladder dysfunction appear, emphasizing the need for comprehensive spinal imaging-including full neuraxis MRI-when TCS is suspected, as incomplete imaging risks missing critical coexisting lesions affecting prognosis and surgery planning. Cardiac anomalies occurred in 21 of the total cases, and among cardiac anomalies, VSD was most common (38.0%), followed by ASD (33.3%) and PDA (23.8%). Few patients had overlapping lesions and features. Complex congenital heart diseases like Tetralogy of Fallot and atrioventricular septal defect were less frequent but clinically significant. The cardiac anomaly rate here slightly exceeds the ~8% reported in earlier TCS studies (e.g., Cunningham et al.) due to improved echocardiographic screening and may also reflect referral bias, where more complex or severe cases are preferentially sent to specialized centers [2]. Developmentally, spinal and cardiac anomalies overlap due to shared embryologic origins during notochord migration. Recent studies suggest TGF-beta disruption in humans and VANGL1/2 & Sonic Hedgehog pathways in animal models underlie both neural tube and cardiac outflow tract defects [21]. Significant overlapping occurred-13.3% had VACTERAL anomaly, 10% had combined gastrointestinal and cardiac anomalies, and 8.3% urinary plus cardiac anomalies, highlighting TCS’s true syndromic nature and the risk of underdiagnosis if screening focuses narrowly. Management of detected cardiac lesions in TCS patients depended on severity: minor defects or valve issues were usually managed conservatively with regular monitoring, while significant lesions required medical therapy (diuretics, ACE inhibitors, beta-blockers) to stabilize heart function before surgery. High-risk lesions were referred to cardiology consultation for possible surgical or catheter-based repair before TCS neurosurgery to reduce perioperative risks. Coordination between cardiology, neurosurgery, and anaesthesiology ensures safe management during surgery and optimal outcomes. During surgery, hemodynamic monitoring and maintenance of adequate mean arterial pressure were critical to prevent spinal cord ischemia and cardiovascular complications. Anaesthetic avoided neuromuscular blockers and volatile agents, when possible, to reduce cardiac stress and preserve neurophysiological monitoring signals. This study supports revising clinical guidelines to include routine echocardiography in TCS assessment. Similarly, all patients should be systemically scanned for renal anomaly using transabdominal ultrasound. Integrating genetic data with clinical findings can also clarify phenotypic variability and familial patterns, thereby enhancing understanding of TCS pathogenesis and informing preventive strategies.

Spectrum of spinal dysraphism

The spectrum of spinal dysraphism demonstrates a varied distribution, with sacral lipomyelomeningocele being the most prevalent type, accounting for 35% of cases, consistent with international data reported by Misbah et al. [22]. These lesions are often diagnosed later due to their subtle clinical presentation. Other forms include meningomyelocele (31.6% combined) and dermal sinus (16.7%), reflecting the developmental heterogeneity of neuraxis closure defects. Rare entities such as diastematomyelia (3.3%), conus dermoids (3.3%), and dorsal meningomyelocele (1.6%) remain uncommon but are clinically significant because their presence increases the risk of infection, recurrent tethering, and poorer outcomes. The lesion distribution in this cohort likely reflects both true prevalence and referral bias, as specialized centers tend to see more complex cases and perform thorough screening. Regional and national data show cardiac anomaly prevalence around 6.3% and spinal anomalies about 24.5% in TCS, slightly lower than the higher rates seen here. Late diagnosis of lipomyelomeningocele correlated with worse outcomes, consistent with evidence that early surgery preserves neurological and bladder function better than delayed intervention. These findings underscore the critical role of high-resolution MRI in thoroughly mapping the extent of spinal dysraphism and are essential for accurate prognostication and management [22].

Surgical management

The surgical management of spinal dysraphism is highly tailored to the lesion type, with procedures like lipoma excision and filum terminale division commonly used for lipomyelomeningocele, and adhesion release prioritized in previously operated meningomyelocele (MMC) patients. The average surgery time was 136 minutes. Early intervention had a good impact on the outcome (p=0.002). Intraoperative findings often reveal a lipoma with a thickened filum terminale in about 33.3% of cases, closely matching preoperative imaging. Patients with prior surgeries, especially those treated with MMC, show a higher risk of adhesions (17.8%), a complication well-known in paediatric populations, including the large series by Ferreira et al. [23]. This highlights the need for meticulous surgical technique, using dura substitutes and intraoperative neuro-monitoring to reduce risks. In our study, intraoperative neuro monitoring was not used as the facility for the same was not available. Overall, radical yet safe detethering that preserves functional neural tissue is essential for neurological recovery. Postoperative complication rates were lower than the literature-reported 10-15% for primary TCS surgeries, as noted by Elmesallamy et al. [24]. Infection and CSF leak risks were higher in complex cases with dermal sinuses. Cardiac complications, such as heart failure (4.4%) and arrhythmia (2.2%), occurred postoperatively only in patients with significant cardiac defects. These findings stress the importance of paediatric cardiology involvement in perioperative management for risk-adjusted anaesthesia in high-risk TCS cases. Long-term complications after TCS surgery extend beyond the immediate postoperative period and include risks such as retethering, neurological deterioration, bladder dysfunction, and pain. There was one episode of retethering. Other long-term urological complications are discussed below.

Follow-up and functional outcomes

A follow-up analysis of 42 patients post-surgery reveals promising trajectories in functional outcomes, highlighting the benefits of timely intervention. The median duration of follow-up was 16 months (range 12-24 months). Urological outcomes showed marked improvement, with 81.25% of patients experiencing significantly better urinary function (p = 0.0106), a result consistent with the largest meta-analyses, which report improvement rates of 60-85% after detethering procedures (Bhimani et al.) [25]. Key urodynamic parameters-including maximum flow rate (Qmax), post-void residual volume, and overall quality of life (QOL)-demonstrated statistically significant enhancements at one-year follow-up in ANOVA analysis (p values ranging from <0.01 to <0.005), paralleling findings by Geyik et al. [26]. Missing data in ANOVA were handled using listwise deletion, where lost to follow-up cases were excluded from analysis. This approach was appropriate because the proportion of missing data was small (n=3) and not expected to introduce bias or reduce statistical power. These improvements were seen to be sustained beyond a one-year follow-up, also. Motor function also improved substantially, with 90% of patients showing recovery of deficits (p approximately 0.01), while bowel dysfunction exhibited a favourable but statistically non-significant improvement in 62.5% of cases. Bowel outcomes showed less significant change post-surgery compared to urinary or motor functions because bowel control involves a more complex network of autonomic and somatic innervation, which may be less responsive to decompression or more prone to irreversible damage if intervention is delayed. Additionally, bowel dysfunction may be influenced by chronicity and overlapping gastrointestinal conditions, reducing sensitivity to neurosurgical effects. Pain and sensory deficits improved in 81.8% and 85.7% of patients, respectively. These data robustly support early surgical intervention before irreversible spinal cord damage ensues. Recurrence of symptoms was rare, noted in two patients each for pain; however, three patients were lost to follow-up, underlining the critical need for comprehensive, long-term patient monitoring. This study also reinforces the role of systematic cardiac screening-including routine echocardiography-as hemodynamically significant but clinically silent cardiac malformations have been documented in up to 10% of neural tube defect cohorts. The newer echocardiographic parameters, such as 4D echo and strain imaging integrated with a new artificial intelligence technique, can enhance the screening sensitivity [27]. Active screening of cardiovascular anomalies in this study did lead to altered management, such as preoperative cardiac optimization and tailored anesthesia protocols, which helped mitigate perioperative risks and stabilize hemodynamics during surgery. However, direct evidence linking these screening results to improved surgical outcomes remains limited due to the small sample size. The findings highlight the indispensability of a multidisciplinary care model, integrating pediatric neurosurgery, urology, cardiology, genetics, and rehabilitation services, with genetic counselling now recognized as best practice in syndromic TCS (Weisbrod et al.) [28]. Rehabilitation after surgery involves gradually resuming activities over 4-6 weeks, emphasizing light exercise such as walking and pelvic floor (Kegel) exercises to support bladder control [29,30]. Enhanced Recovery After Surgery is a multidisciplinary, evidence-based protocol designed to reduce surgical stress and accelerate recovery, ultimately improving postoperative outcomes and minimizing distress [30]. Finally, addressing the persistent challenge of re-tethering, this cohort’s experience supports Pan et al.'s recommendations for employing novel intraoperative adjuncts such as ultrasound and neurophysiological monitoring to decrease morbidity and improve surgical outcomes [31].

Study strengths and limitations

This study has several strengths and some limitations. Prospective data collection minimized recall bias. Comprehensive cardiac screening improved the detection of hidden heart issues. Use of standardized tests, including urodynamic studies and quality-of-life assessments, ensured reliable and comparable results. This study’s higher prevalence of cardiovascular and other anomalies may reflect referral bias, as more complex TCS cases are sent to specialized centers. Its single-center design and small sample size limit generalizability. A key limitation is the lack of genetic or molecular testing, which is important for syndromic associations like VACTERL and could aid in identifying underlying causes and better patient stratification. Socioeconomic and healthcare access challenges may also have led to delayed presentation and follow-up. While the study offers valuable insights, larger multicenter research with genetic assessment is needed to validate and extend these findings.

## Conclusions

This study demonstrates significant improvements following TCS surgery, with many patients regaining urinary function and showing marked motor recovery. Bowel function also improved in a notable proportion of cases, while recurrence and adhesion rates remained low and comparable to existing literature. Routine cardiac screening revealed previously undetected heart anomalies, enabling safer perioperative management. Multidisciplinary follow-up, including renal ultrasound and urodynamic studies, facilitated early detection of complications and contributed to better long-term outcomes. Genetic studies targeting relevant genes are still underutilized but hold potential for enhancing risk assessment and guiding recovery strategies. Overall, these findings emphasize the importance of comprehensive cardiac evaluation and multidisciplinary care in optimizing outcomes for TCS patients.
